# Dental implant removal rates and related factors in older adults in Korea: a cross-sectional study using National Health Insurance Sharing Service database

**DOI:** 10.4178/epih.e2025064

**Published:** 2025-12-03

**Authors:** Hyang-Ah Park, A-Rang Lim, Jae-In Ryu

**Affiliations:** 1 Department of Preventive and Social Dentistry, Kyung Hee University College of Dentistry, Seoul, Korea; 2 Department of Dental Hygiene, Bucheon University, Bucheon, Korea; 3 Department of Preventive and Social Dentistry, Graduate School, Kyung Hee University, Seoul, Korea

**Keywords:** Dental implants, Aged, Health insurance, Cohort studies, Health services for the aged, Risk factors

## Abstract

**OBJECTIVES:**

Dental implants have been covered by the National Health Insurance for older adults in Korea since 2014. As the number of implant treatments has increased, the occurrence of related complications has also grown. While many clinical studies have examined mechanical and biological complications, few epidemiological studies using national data have identified population-level risk factors. This study aimed to determine the dental implant removal rate and related factors among older adults based on the Andersen Behavioral Model.

**METHODS:**

A cross-sectional analysis was conducted using the National Health Insurance Service–National Sample Cohort. Logistic regression analyses were performed to identify factors associated with implant removal. Unadjusted and stepwise cumulative models were applied for predisposing, enabling, and need factors. All analyses were conducted using SPSS version 26.0, with statistical significance set at α=0.05.

**RESULTS:**

The overall dental implant removal rate among older adults was approximately 0.4%. Higher removal rates were observed among males, those aged 65-69, current smokers, individuals with diabetes, and the self-employed group with higher income levels. Current smokers had 1.75 times higher odds of implant removal (95% confidence interval, 1.39 to 2.22) than non-smokers or former smokers.

**CONCLUSIONS:**

Targeted education, management, and preventive interventions for high-risk groups, including smokers and patients with diabetes, are necessary to improve implant success and reduce complications. These findings provide population-based evidence to inform public health strategies that promote successful implant outcomes among older adults.

## GRAPHICAL ABSTRACT

[Fig f1-epih-47-e2025064]

## Key Message

This study conducted a multifactorial analysis based on the Andersen model to identify factors associated with dental implant removal using national big data, addressing the lack of prior studies that examined these determinants sufficiently. The dental implant removal rate among all subjects was approximately 0.4%, and higher removal rates were observed among males, younger individuals, the self-employed group with higher income quintiles, and individuals with diabetes, smoking habits, or engagement in aerobic physical activity. Current smokers had a 1.8-fold higher probability of implant removal. These findings reinforce the importance of developing and implementing guidelines to increase the success rate of dental implants.

## INTRODUCTION

The proportion of older adults (those aged 65 or above) is expected to increase rapidly from 10% in 2022 to 16% in 2050 [1]. Older people often have several chronic diseases such as hearing loss, chronic obstructive pulmonary disease, osteoarthritis, and diabetes [2]. They also experience a 3-fold to 5-fold higher prevalence of a “poor” subjective health status or limitations in activities of daily living than others [3]. Oral health can similarly change with age, including decreased saliva secretion or reduced oral care ability [4-6], along with increased tooth loss [7]. The edentulous rate is 20 times higher in older adults than in individuals aged 20-44 [8]. It is therefore important to rehabilitate tooth loss with dental prostheses in a timely manner, particularly among older adults. Fixed prostheses were previously used more commonly; however, they have low patient satisfaction [9]. Removable dentures can cause dislodgement, poor support and stability, and pain [10]. As a result, demand for dental implants, which can minimize these disadvantages, has continued to increase.

The National Health Insurance in Korea introduced dental implants into the coverage system in 2014, becoming the first country to do so [11]. Coverage began with 50% out-of-pocket expenses for adults aged 75 and older. Since 2018, the eligible age has expanded to 65 years and older, with the out-of-pocket cost lowered to 30%. Consequently, the number of patients receiving dental implant procedures increased rapidly from 52,873 in 2014 to 1,776,827 in 2022, a 33.6-fold increase [12]. Paradoxically, the occurrence of dental implant-related complications has also risen alongside expanded coverage. Removal surgeries for dental implants increased from 2,418 in 2014 to 85,403 in 2022, a 35.3-fold increase. Legal disputes related to dental implants have grown as well, accounting for 38.8% of all claims received by insurance companies. The most common issues were nerve damage (18.7%), implant failure (13.5%), and peri-implantitis (6.5%) [13].

Dental implants involve both surgical and prosthetic components. A range of complications can occur during or after implant surgery or prosthesis installation. These complications are broadly categorized into mechanical and biological types [14,15]. Among mechanical complications, abutment screw loosening, prosthesis dislodgement due to loss of retention, and ceramic material fracture in the prosthesis after 5 years accounted for approximately 8.8%, 4.1%, and 3.5% of cases, respectively [16]. These complications may be influenced by factors such as implant diameter, length, and placement location [17,18]. Regarding biological complications, peri-implant mucositis—a reversible inflammatory reaction of the mucosa surrounding the implant—and peri-implantitis, an inflammatory reaction associated with bone resorption, are commonly reported with long-term use [19,20]. Peri-implantitis presents with clinical features such as bleeding on probing, attachment or bone loss greater than 2.5 mm, and periodontal pocket depth exceeding 6 mm [21]. Although numerous studies have examined mechanical and biological complications of dental implants, relatively few epidemiological studies have been conducted. The factors related to implant complications remain unclear. Some studies have indicated that dental implant outcomes may be affected by socioeconomic factors [22]. Surgical complications such as infection and delayed healing may be more common in patients with diabetes who have poor glycemic control [23]. Smoking has also been identified as a major factor contributing to implant failure [24]. Therefore, research to identify the diverse factors associated with dental implant failure is increasingly necessary. However, studies using national big-cohort data remain insufficient.

Thus, this study aimed to determine the status of dental implant removal rates among Korean seniors since the introduction of national insurance coverage in 2014 and to identify related factors based on the Andersen Behavioral Model.

## MATERIALS AND METHODS

### Study subjects

The National Sample Cohort Database, a claims-based database from the National Health Insurance Sharing Service, was used to analyze the status of dental implant removal and related factors after reimbursement. The National Sample Cohort is a research database covering the years 2002 to 2019 and includes approximately 1 million people, representing 2% of the Korean population. This database contains socioeconomic information (such as sex, age, health insurance type, income quintile, and region), treatment records, and medical resource utilization data (including consultations and medical checkups). For analyses of the dental implant removal rate, the most recent data following reimbursement from 2018 to 2019 were used. In total, the number of subjects aged over 65 years in this cohort was 325,835.

### Study variables

According to the Andersen Behavioral Model, used as an interpretive framework for healthcare utilization, factors related to dental implant removal were categorized into predisposing, enabling, and need factors. Predisposing factors consisted of sex and age. Age was categorized into 65-69 years, 70-74 years, and 75 years or older. Enabling factors consisted of residential area, insurance type, and income quintiles. Residential areas were categorized into metropolitan areas and provinces. Insurance types were classified as self-employed, employee, and Medical Aid. Income quintiles were reclassified into 2 groups, 1-5 and 6-10, for both the self-employed and employee categories. Need factors consisted of disability, hypertension, diabetes, high-risk drinking, current smoking, and aerobic physical activity. Disability and aerobic physical activity were included because they represent evaluated health conditions that influence healthcare utilization. Disability reflects functional limitations, whereas aerobic physical activity represents behavioral and physiological needs related to health maintenance. For health status and behavioral data, the health checkup database was used. Health checkups were offered once every 2 years for office workers and once a year for non-office workers. Thus, 2 continuous years were grouped as 1 period. For non-office workers who received 2 checkups within a period, data from the earlier year were used. People with high-risk drinking were defined as those who consumed alcohol twice or more a week and had an intake of over 7 drinks for males and 5 drinks for females on a single occasion. Current smokers were defined as individuals who were smoking at the time of assessment and had smoked more than 100 cigarettes in their lifetime. Active physical activity was defined as engaging in more than 2 hours and 30 minutes of moderate-intensity physical activity per week or 1 hour and 15 minutes of vigorous-intensity physical activity per week. When participants engaged in both types of activity, 1 minute of vigorous-intensity activity was converted to 2 minutes of moderate-intensity activity. The dental implant removal rate, the outcome variable, was defined as the percentage of people who claimed simple or complex dental implant removal surgery using the codes U4981 or U4982.

### Statistical analysis

Cross-tabulated distributions and the chi-square test were used to examine the relationship between dental implant removal and related factors based on Andersen’s Behavioral Model. Logistic regression analysis was then conducted to evaluate these associations after adjusting for the influence of variables within each factor. An individual variable model (unadjusted) and a stepwise cumulative model (models 1, 2, and 3) were applied to account for the hierarchical influence of predisposing, enabling, and need factors. To reduce potential multicollinearity and overlapping explanatory power among socioeconomic variables, a stepwise modeling approach was used, and income was reclassified by insurance type (lower: quantiles 1-5; higher: quantiles 6-10) to reflect different income structures and maintain analytical stability. Variance inflation factors (VIFs) were used to assess multicollinearity among socioeconomic variables. VIF>10 indicated the presence of multicollinearity [25]. However, no evidence of multicollinearity was identified. All analyses were performed using SPSS version 26.0 (IBM Corp., Armonk, NY, USA), with statistical significance set at α=0.05.

### Ethics statement

The study protocol was approved by the Institutional Review Board (IRB) of Kyung Hee University (IRB No. KHSIRB-23-446 [EA]). Informed consent was waived by the IRB. This retrospective dataset of the national sample cohort did not contain personally identifiable information. All methods were carried out following the National Health Insurance Sharing Service analytic guidelines and regulations.

## RESULTS

There were a total of 325,835 subjects aged over 65 years (Table 1). Most participants were females, aged 75 years or older, residing in a province, classified as employees, and in the higher income quintiles. The proportions of individuals with disability, hypertension, diabetes, high-risk drinking, current smoking, and no aerobic physical activity were higher than those exhibiting the opposite patterns. The overall dental implant removal rate was 0.39%. Among the predisposing factors, sex and age showed statistically significant differences, with higher removal rates observed in males and individuals aged 65-69 years. Within the enabling factors, insurance type demonstrated a stepwise decrease in removal rates from self-employed to employee to Medical Aid beneficiaries. Higher income quintiles among the self-employed group were also associated with increased implant removal rates. Among the need factors, those with diabetes, those who currently smoked, and those engaging in aerobic physical activity exhibited higher removal frequencies.

### Logistic regression for risk factors associated with dental implant removals in older adults

The logistic regression analysis for each factor showed results consistent with the crosstab analysis. The likelihood of experiencing dental implant removal was higher in males and younger individuals based on predisposing factors (Table 2). The odds were 2.12 times higher in those aged 70-74 compared with those aged 75 or older. Regarding enabling factors, individuals living in metropolitan areas or classified as health insurers had a higher probability of implant removal. Differences by insurance type were substantial, with health insurers exhibiting removal rates 3 times higher than those receiving Medical Aid. Among the need factors, the probability of removal was higher in those without a disability, those with diabetes, current smokers, and those who frequently engaged in aerobic physical activity. Notably, current smokers had a 2.65-fold higher probability of implant removal than non-smokers. The difference between model 1, which was adjusted only for predisposing factors, and model 2, which was adjusted for predisposing and enabling factors, was not substantial. However, in model 3, which included all factors, statistically significant associations for disability and diabetes were no longer observed. Logistic regression analysis was also conducted separately for the self-employed and employee groups using the fully adjusted model 3 (Table 3). Among the self-employed, the likelihood of dental implant removal was higher in males and younger individuals. For enabling factors, higher insurance payers had a greater probability of removal. For need factors, removal likelihood was higher in individuals without hypertension, current smokers, or those engaging in aerobic physical activity. For example, current smokers had an approximately threefold higher likelihood of implant removal. In the employee group, the probability of removal was higher among males and individuals aged 65-69 years regarding predisposing factors; among those living in metropolitan areas regarding enabling factors; and among those engaging in high-risk drinking or aerobic physical activity regarding need factors.

## DISCUSSION

As global concerns regarding healthy aging continue to grow, diverse measures are being implemented to support older adults [2,26]. Because Korea is rapidly transitioning into an aging society, the need for universal health coverage for older adults has become increasingly important [27]. Dental implants were first covered by national health insurance in 2014, marking the first such case worldwide [11]. Since then, the number of dental implant procedures has risen substantially, but the frequency of their removal has also increased [12]. This study analyzed risk factors associated with dental implant removal using national cohort data and applied the Andersen Behavioral Model as an overarching framework. Although several risk factors identified in this study—such as sex, age, smoking, and diabetes—have been consistently reported in prior research [28,29], the present analysis extends the existing evidence by offering a population-level interpretation within the Andersen framework [30]. Through this approach, socioeconomic and behavioral dimensions were systematically incorporated, illustrating implant removal not only as a biological outcome but also as a reflection of healthcare access and structural inequality. This provides a broader public health perspective beyond traditional clinical interpretations.

Among the predisposing factors in the fully adjusted model, the probability of dental implant removal among males was 1.65 times higher than that among females. Previous studies likewise reported that males experience dental implant failure more frequently than females [28,29]. One explanation is that males generally have a higher risk of severe periodontitis, which can progress to peri-implantitis and increase the likelihood of implant failure [31]. Therefore, tailored educational programs and management strategies targeting males may be beneficial to support better periodontal self-care. In this study, individuals aged 65-69 years also had a higher likelihood of implant removal. Adults aged over 75 years often wear partial or full dentures rather than implants because they have experienced extensive tooth loss [32]. Implant procedures are more likely to be performed when expected success rates are sufficiently high for these patients as well [33]. Numerous studies have reported that chronological age alone is rarely a direct risk factor for implant failure [34,35]. Instead, multifactorial or derivative age-related factors may contribute to risk. Thus, efforts to reduce implant failure should focus on age-associated conditions rather than age itself during clinical decision-making.

Among the enabling factors, the gap in implant removal rates according to insurance type was the largest. Removal rates were 0.46% for the self-employed, 0.39% for employees, and 0.13% for individuals receiving Medical Aid. The likelihood of implant removal was 2.82 times higher for the self-employed and 2.51 times higher for employees compared with those receiving Medical Aid. This pattern may reflect differences in economic burden: self-employed and employee groups bear higher out-of-pocket expenses than Medical Aid beneficiaries, which may affect implant use and follow-up care [36]. Among the self-employed, individuals in higher income quartiles had a 2.36-fold higher likelihood of implant removal, whereas no significant gap appeared in the employee group. Despite insurance coverage, dental implant utilization remains strongly influenced by personal income, with higher-income adults using implants more frequently [37]. Income among self-employed older adults may play a relatively stronger role in their decision to undergo implant surgery. When a procedure is carried out, the removal rate may be similar because the initial decision to proceed is already economically driven. Many self-employed older adults in Korea have lower income levels and poorer health status because they are unemployed or operating small or unstable businesses [38,39]. Large income disparities may therefore influence treatment outcomes. Future research should clarify whether the characteristics of being self-employed exert direct effects on implant removal or whether indirect socioeconomic mechanisms are implicated.

Current smoking status within the need factors emerged as an important risk factor for implant removal, with smokers showing approximately 1.8 times higher odds than non-smokers or former smokers. Smoking interferes with osseointegration during the early healing phase after implantation and is therefore strongly associated with early implant failure [39,40]. Both the presence and amount of smoking are closely linked to implant outcomes, underscoring the need for active interventions targeting smoking behavior [41-43]. Moreover, the likelihood of implant removal for current smokers was greater among the self-employed, at 2.25 times higher, compared with 1.59 times higher among employees. These findings suggest that self-employed insurance beneficiaries may be an especially important priority group for smoking cessation and oral health interventions.

Diabetes has a substantial impact on dental implant removal. Patients with diabetes whose blood sugar levels are not properly controlled often experience delayed healing, which may lead to early implant failure [44]. Peri-implantitis and peri-implant mucositis also occur more frequently in this population because of weakened immune responses, which contribute to biological factors associated with late implant failure [45,46]. Preemptive intervention and management may therefore be necessary when placing dental implants in patients with diabetes to reduce the risk of failure. However, in this study, statistically significant associations for diabetes were inconsistent, with both significant and non-significant findings across models. Thus, continued follow-up is required to clarify this risk.

Meanwhile, individuals with higher income levels among the self-employed and those engaging in aerobic physical activity showed higher removal rates within these groups. This finding suggests that these factors do not solely reflect biological risk but instead represent socioeconomic conditions, variations in healthcare accessibility, utilization behavior, and opportunities to receive dental implant procedures. Individuals with lower socioeconomic status may receive dentures rather than implants, resulting in fewer cases where implant removal could occur. Therefore, it is important to adopt a structural perspective that considers inequalities in service utilization and access, rather than attributing differences entirely to biological vulnerability.

This study has some limitations. First, dental implant removal rates from 2014 to 2019 were used. However, it was not possible to determine whether implants were removed after coverage began, requiring caution when interpreting removal as a direct consequence of reimbursement. Second, the study used National Health Insurance Service claims data from local dental clinics. Because dental implantation is a relatively expensive procedure, dentists may remove failed implants without claiming the procedure to avoid charging patients. As a result, there is a high possibility of missing removal cases that were provided but not billed. To address these limitations, future research should adopt a cohort design that follows individuals from placement to removal and performs in-depth longitudinal analyses. Third, the claim codes in Korea only specify implant placement and removal [47]. In clinical practice, many mechanical and biological complications occur between these 2 procedures [48-50]. Therefore, it is necessary to investigate the prevalence and determinants of these intermediate complications, as was done in this study for implant removals, to further improve implant success. Fourth, the regression model coefficients in this study may be low. A more sophisticated study design incorporating additional relevant factors will be necessary in future research. Some health status indicators, such as body mass index, serum cholesterol, and the Charlson comorbidity index, could not be fully included because of high missing rates, inconsistent measurement, or insufficient case numbers. Instead, diabetes, hypertension, and disability registration were used as proxy variables to represent systemic health. Finally, this study was descriptive and based on the Andersen Behavioral Model, emphasizing structural interpretation rather than causal inference. Stratified analyses by insurance type supplemented this approach by showing population-level variations. Despite these limitations, this study is meaningful because it examined dental implant removal and related factors using nationally representative data.

According to the analysis using Andersen’s model of dental implant removal in older adults in Korea, removal rates were higher among males in terms of predisposing factors and among the self-employed in terms of enabling factors. Targeted education, management, and active intervention before and after implant placement should therefore be prioritized for these groups. For current smokers and patients with diabetes—key at-risk groups—the likelihood of implant removal increases. These conditions should be identified prior to implant placement to ensure preemptive intervention and management. Ultimately, recommendations based on these findings are essential to improving dental implant success and supporting evidence-based dentistry.

## Figures and Tables

**Figure f1-epih-47-e2025064:**
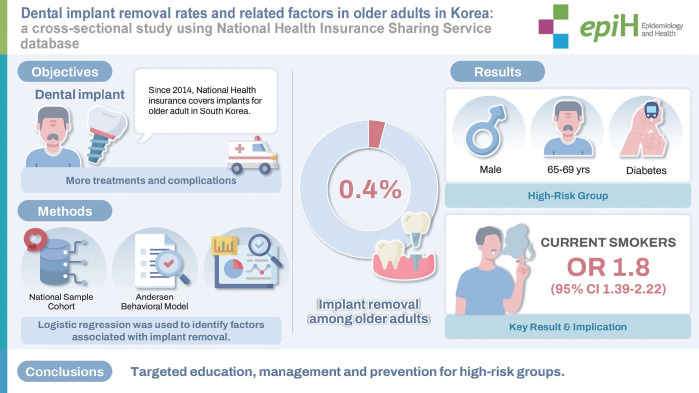


**Table 1.** General characteristics and implant removal rates among individuals aged 65 years or older

Factor type	Variables	Total	Implant removal rate	p-value
Total		325,835 (100)	1,277 (0.39)	
Predisposing	Sex			<0.001
	Female	185,187 (56.8)	519 (0.28)	
	Male	140,648 (43.2)	758 (0.54)	
	Age (yr)			<0.001
	≥75	145,488 (44.7)	384 (0.26)	
	70-74	79,398 (24.4)	392 (0.49)	
	65-69	100,949 (31.0)	501 (0.50)	
Enabling	Location of residence			<0.001
	Province	187,895 (57.7)	663 (0.35)	
	Metropolitan	137,940 (42.3)	614 (0.45)	
	Insurance type			<0.001
	Medical Aid	20,874 (6.4)	27 (0.13)	
	Self-employed	92,624 (28.4)	422 (0.46)	
	Employee	212,337 (65.2)	828 (0.39)	
	Income quantiles			
	Self-employed			<0.001
	1-5	33,197 (35.8)	72 (0.22)	
	6-10	59,405 (64.2)	349 (0.59)	
	Employee			0.560
	1-5	68,224 (32.9)	265 (0.39)	
	6-10	139,444 (67.1)	540 (0.39)	
Needs	Disability			0.005
	Yes	53,733 (16.5)	173 (0.32)	
	No	272,102 (83.5)	1,104 (0.41)	
	Hypertension			0.378
	No	223,158 (68.5)	860 (0.39)	
	Yes	102,677 (31.5)	417 (0.41)	
	Diabetes			0.001
	No	284,024 (87.2)	1,072 (0.38)	
	Yes	41,811 (12.8)	205 (0.49)	
	Current smoking			<0.001
	No	316,025 (97.0)	1,186 (0.38)	
	Yes	9,810 (3.0)	91 (0.93)	
	High-risk drinking			0.134
	No	322,594 (99.0)	1,259 (0.39)	
	Yes	3,241 (1.0)	18 (0.56)	
	Aerobic physical activity			<0.001
	No	228,807 (70.2)	742 (0.32)	
	Yes	97,028 (29.8)	535 (0.55)	

Values are presented as number (%).

**Table 2.** Logistic regression analysis of dental implant removal rates in individuals aged 65 years or older

Factor type	Variables	Unadjusted	p-value	Fully adjusted					
				Model 1	p-value	Model 2	p-value	Model 3	p-value
Predisposing	Sex								
	Female	1.00 (reference)		1.00 (reference)		1.00 (reference)		1.00 (reference)	
	Male	1.91 (1.70, 2.15)	<0.001	1.79 (1.59, 2.02)	<0.001	1.77 (1.57, 1.99)	<0.001	1.65 (1.46, 1.86)	<0.001
	Age (yr)								
	≥75	1.00 (reference)		1.00 (reference)		1.00 (reference)		1.00 (reference)	
	70-74	2.12 (1.83, 2.46)	<0.001	2.01 (1.73, 2.33)	<0.001	1.95 (1.68, 2.26)	<0.001	1.80 (1.55, 2.09)	<0.001
	65-69	2.05 (1.78, 2.36)	<0.001	1.93 (1.67, 2.22)	<0.001	1.85 (1.61, 2.14)	<0.001	1.67 (1.45, 1.94)	<0.001
Enabling	Location of residence								
	Province	1.00 (reference)		-		1.00 (reference)		1.00 (reference)	
	Metropolitan	1.27 (1.13, 1.42)	<0.001	-		1.22 (1.09, 1.37)	0.001	1.20 (1.07, 1.35)	0.002
	Insurance type								
	Medical Aid	1.00 (reference)		-		1.00 (reference)		1.00 (reference)	
	Self-employed	3.48 (2.32, 5.22)	<0.001	-		2.98 (1.99, 4.48)	<0.001	2.82 (1.88, 4.24)	<0.001
	Employee	3.10 (2.08, 4.62)	<0.001	-		2.70 (1.81, 4.03)	<0.001	2.51 (1.68, 3.74)	<0.001
Needs	Disability								
	Yes	1.00 (reference)		-		-		1.00 (reference)	
	No	1.24 (1.05, 1.47)	0.010	-		-		1.11 (0.93, 1.31)	0.244
	Hypertension								
	No	1.00 (reference)		-		-		1.00 (reference)	
	Yes	1.09 (0.96, 1.23)	0.193	-		-		0.88 (0.77, 1.00)	0.054
	Diabetes								
	No	1.00 (reference)		-		-		1.00 (reference)	
	Yes	1.35 (1.15, 1.57)	<0.001	-		-		1.17 (1.00, 1.38)	0.055
	High-risk drinking								
	No	1.00 (reference)		-		-		1.00 (reference)	
	Yes	1.50 (0.91, 2.46)	0.111	-		-		0.87 (0.53, 1.43)	0.577
	Current smoking								
	No	1.00 (reference)		-		-		1.00 (reference)	
	Yes	2.65 (2.11, 3.33)	<0.001	-		-		1.75 (1.39, 2.22)	<0.001
	Aerobic physical activity								
	No	1.00 (reference)		-		-		1.00 (reference)	
	Yes	1.78 (1.58, 2.00)	<0.001	-		-		1.42 (1.25, 1.60)	<0.001
Nagelkerke R^2^				0.018		0.021		0.026	

Values are presented as odds ratio (95% confidence interval).

**Table 3.** Logistic regression analysis of dental implant removal rates in individuals aged 65 years or older by insurance type

Factor type	Variables	Self-employed				Employee			
		Unadjusted	p-value	Fully adjusted	p-value	Unadjusted	p-value	Fully adjusted	p-value
Predisposing	Sex								
	Female	1.00 (reference)		1.00 (reference)		1.00 (reference)		1.00 (reference)	
	Male	2.05 (1.66, 2.54)	<0.001	1.66 (1.34, 2.07)	<0.001	1.83 (1.58, 2.11)	<0.001	1.64 (1.41, 1.91)	<0.001
	Age (yr)								
	≥75	1.00 (reference)		1.00 (reference)		1.00 (reference)		1.00 (reference)	
	70-74	2.14 (1.66, 2.83)	<0.001	1.70 (1.29, 2.23)	<0.001	2.03 (1.69, 2.43)	<0.001	1.80 (1.49, 2.17)	<0.001
	65-69	1.84 (1.42, 2.37)	<0.001	1.41 (1.08, 1.84)	0.011	2.07 (1.74, 2.46)	<0.001	1.77 (1.48, 2.13)	<0.001
Enabling	Location of residence								
	Province	1.00 (reference)		1.00 (reference)		1.00 (reference)		1.00 (reference)	
	Metropolitan	1.19 (0.79, 1.47)	0.092	1.08 (0.88, 1.33)	0.448	1.32 (1.14, 1.52)	<0.001	1.24 (1.08, 1.44)	0.003
	Income quantiles								
	1-5	1.00 (reference)		1.00 (reference)		1.00 (reference)		1.00 (reference)	
	6-10	2.78 (2.13, 3.64)	<0.001	2.36 (1.79, 3.09)	<0.001	0.96 (0.83, 1.12)	0.628	1.16 (0.99, 1.36)	0.061
Needs	Disability								
	Yes	1.00 (reference)		1.00 (reference)		1.00 (reference)		1.00 (reference)	
	No	1.35 (0.98, 1.87)	0.070	1.17 (0.84, 1.63)	0.342	1.15 (0.94, 1.40)	0.181	1.05 (0.86, 1.29)	0.617
	Hypertension								
	No	1.00 (reference)		1.00 (reference)		1.00 (reference)		1.00 (reference)	
	Yes	1.00 (0.80, 1.25)	0.975	0.78 (0.61, 0.99)	0.041	1.08 (0.94, 1.26)	0.284	0.93 (0.79, 1.09)	0.387
	Diabetes								
	No	1.00 (reference)		1.00 (reference)		1.00 (reference)		1.00 (reference)	
	Yes	1.43 (1.09, 1.88)	0.011	1.28 (0.96, 1.71)	0.094	1.30 (1.07, 1.57)	0.008	1.14 (0.93, 1.40)	0.213
	High-risk drinking								
	No	1.00 (reference)		1.00 (reference)		1.00 (reference)		1.00 (reference)	
	Yes	0.85 (0.27, 2.67)	0.784	0.50 (0.16, 1.56)	0.229	1.76 (1.01, 3.06)	0.045	1.11 (0.64, 1.94)	0.713
	Current smoking								
	No	1.00 (reference)		1.00 (reference)		1.00 (reference)		1.00 (reference)	
	Yes	2.99 (2.01, 4.45)	<0.001	2.25 (1.49, 3.38)	<0.001	2.38 (1.79, 3.17)	<0.001	1.59 (1.18, 2.15)	0.002
	Aerobic physical activity								
	No	1.00 (reference)		1.00 (reference)		1.00 (reference)		1.00 (reference)	
	Yes	1.89 (1.54, 2.33)	<0.001	1.51 (1.21, 1.89)	<0.001	1.65 (1.43, 1.90)	<0.001	1.32 (1.13, 1.54)	0.001
Nagelkerke R^2^		0.039				0.020			

Values are presented as odds ratio (95% confidence interval).
